# Effectiveness of Balance- and Strength-Based Exercise Interventions for Fall Prevention in Community-Dwelling Older Adults: A Systematic Review of Randomized Controlled Trials

**DOI:** 10.3390/life16010041

**Published:** 2025-12-26

**Authors:** Prashant Kumar Choudhary, Suchishrava Choudhary, Sohom Saha, Borko Katanić, İsmail İlbak, Gusztáv József Tornóczky

**Affiliations:** 1 Department of Physical Education Pedagogy, Lakshmibai National Institute of Physical Education, Shakti Nagar, Race Course Road, Gwalior 474002, India; prashantlnipe2014@gmail.com; 2 Department of Sport Psychology, Lakshmibai National Institute of Physical Education, Shakti Nagar, Race Course Road, Gwalior 474002, India; suchishrava05@gmail.com (S.C.); sohomsaha77@gmail.com (S.S.); 3 Montenegrin Sports Academy, 81000 Podgorica, Montenegro; borkokatanic@gmail.com; 4 Institute of Health Sciences, İnönü University, Malatya 44050, Türkiye; isma_ilbak@hotmail.com; 5 Institute of Health Promotion and Sport Sciences, Faculty of Education and Psychology, ELTE Eötvös Loránd University, 1075 Budapest, Hungary; 6 Physical Education and Exercise Centre, Medical School, University of Pécs, 7624 Pécs, Hungary

**Keywords:** aging population, balance training, strength training, mobility, reactive balance, fall risk reduction

## Abstract

Background: Falls are a leading cause of injury, disability, and loss of independence among community-dwelling older adults. Although exercise-based interventions are widely recommended for fall prevention, the comparative effectiveness of different exercise modalities remains insufficiently synthesized. Methods: This systematic review was conducted in accordance with PRISMA 2020 guidelines. Randomized and controlled trials were identified through searches of PubMed, Scopus, Web of Science, and CINAHL, including studies involving adults aged ≥60 years who participated in balance-based, strength-based, multimodal, Tai Ji Quan, Otago Exercise Program, or perturbation-based reactive balance training interventions. Methodological quality was assessed using the Cochrane Risk of Bias 2 (ROB-2) tool. Due to substantial clinical and methodological heterogeneity across interventions and outcome measures, a narrative synthesis was undertaken. Results: Twenty-seven trials met the inclusion criteria. Exercise interventions consistently reduced fall incidence across studies. Tai Ji Quan interventions were associated with approximately 31–58% reductions in falls, the Otago Exercise Program with 23–40% reductions, and multimodal strength–balance training with 20–45% reductions. Perturbation-based reactive balance training demonstrated particularly strong effects on laboratory-induced falls, with reductions ranging from 50–75%. Functional outcomes also improved across intervention types, including faster Timed Up and Go performance, increased gait speed, improvements of approximately 1.2–2.5 points in Short Physical Performance Battery scores, 15–35% gains in lower-limb strength, and enhanced reactive balance responses. Longer-duration interventions (12–24 months) generally demonstrated sustained reductions in fall risk. Conclusions: Evidence from randomized and controlled trials indicates that structured exercise interventions, particularly Tai Ji Quan, the Otago Exercise Program, multimodal strength–balance training, and perturbation-based reactive balance training, are effective in reducing falls and improving balance, mobility, and strength in community-dwelling older adults. These findings support the use of targeted, evidence-based exercise programs as central components of fall-prevention strategies in older populations.

## 1. Introduction

Falls remain a major threat to the health and independence of older adults, with nearly one in three individuals aged ≥65 years experiencing at least one fall annually. Beyond physical injury, fear of falling is a clinically relevant factor strongly associated with balance impairment, activity restriction, and functional decline. Recent evidence demonstrates significant correlations between fear of falling scores and objective measures of functional balance in community-dwelling older adults, supporting its role as a valid indicator of fall risk [1]. Recent global estimates indicate that falls account for over 684,000 deaths annually worldwide, with adults aged 60 years and older disproportionately affected. The World Health Organization reports that fall constitute the second most common cause of death due to unintentional injuries across the globe, highlighting their significant global impact [2]. Additional epidemiological data suggest that around 40% of community-dwelling adults over 65 years encounter at least one fall each year, often resulting in injury, functional deterioration, and diminished overall well-being [3]. In the European context, fall-related fatalities among older adults are notably high, with approximately 36,000 deaths recorded annually; notably, individuals aged 75 years and older account for the majority of these cases (88%), and women represent a disproportionately affected group (59%) [4]. Collectively, these findings underscore the critical importance of developing and implementing effective fall-prevention strategies for aging populations.

Physical inactivity and reductions in functional capacity, such as weakened lower-limb muscles and compromised balance, are well-established risk factors contributing to falls and fall-related injuries, including head trauma and hip fractures, among older adults [5,6,7,8]. Participating in sports and physical activity not only shapes the human body but also offers a transformative experience that enhances both physical and mental well-being, further reinforcing its value as a preventive health strategy [9,10]. Although these physiological deficits significantly elevate fall vulnerability, they remain highly modifiable through targeted exercise interventions [6,11,12]. Beyond their physical consequences, falls create substantial financial strain due to increased medication use, hospital admissions, and extended rehabilitation services [2,13]. A robust body of evidence supports exercise training as one of the most effective approaches for enhancing functional performance, improving mobility and balance, and ultimately reducing fall risk in older adults [14,15,16]. Additionally, promoting regular physical activity yields considerable economic and public health benefits, contributing positively to national healthcare systems and improving quality of life at both community and individual levels [17].

Given the substantial individual, societal, and economic burden associated with falls, researchers and clinicians have increasingly emphasized the importance of identifying modifiable factors that can effectively mitigate fall risk among older adults [18,19]. A considerable body of scientific literature has demonstrated that deficits in physical fitness, including decreased muscle strength, slower gait speed, impaired balance, and poor postural control, directly contribute to fall likelihood and fall-related injuries [5,6,8]. These impairments tend to worsen progressively with age, particularly in adults over 70 years, as natural age-related declines in neuromuscular function accelerate deterioration in reaction time, proprioception, and coordination. Importantly, these deteriorations are not merely a consequence of aging, but are significantly exacerbated by Sedentary lifestyles and insufficient participation in structured physical activity [7]. Therefore, maintaining adequate levels of physical activity becomes a central component in preventing early functional decline in older populations.

The evidence linking functional decline with fall risk is further reinforced by longitudinal studies indicating that lower-limb strength is one of the strongest predictors of fall likelihood, particularly in tasks requiring rapid balance recovery or directional changes [11]. Weakness in key muscle groups such as the quadriceps, gluteals, and ankle stabilizers reduces the capacity to execute compensatory steps or prevent center-of-mass displacement during unexpected perturbations [20,21]. Similarly, impaired balance abilities, often measured through standardized assessments such as the Timed Up and Go (TUG) or Berg Balance Scale (BBS), are strongly associated with increased fall incidence, highlighting the role of neuromuscular control in stabilizing the body during dynamic tasks [12,16]. Combining these findings, the literature consistently underscores that physical inactivity sets off a cascade of functional deterioration, which in turn contributes to a heightened risk of falls and fall-induced injuries.

In addition to physiological risk factors, several behavioral and environmental contributors have been identified. Poor health behaviors, lack of engagement in wellness-promoting activities, and low adherence to exercise routines all independently elevate fall risk [7]. Environmental hazards such as poor lighting, uneven flooring, and cluttered home environments also contribute, especially among older adults with limited mobility and slower reflexes. Nevertheless, while environmental and behavioral factors play important roles, interventions targeting physical function, particularly those involving structured exercise, remain among the most effective and sustainable strategies for preventing falls. This is supported by systematic reviews showing that exercise-based interventions lead to consistent improvements in muscle strength, functional mobility, balance control, and gait stability, which collectively translate into measurable reductions in fall incidence [14].

From a biomechanical perspective, fall risk in older adults reflects the interaction between lower-limb muscle strength, postural control, and reactive balance capacity. Age-related weakness in key muscle groups and impaired anticipatory control reduce the ability to regulate the center of mass and execute effective balance-recovery responses during daily activities. When unexpected perturbations such as slips or trips occur, insufficient reactive balance further limits compensatory stepping, increasing the likelihood of falls.

The growing emphasis on exercise interventions is further justified by robust economic evidence. Falls are associated with high medical expenditures, including emergency care, orthopedic surgeries, extended hospital stays, and long-term rehabilitation services [13]. At the societal level, these costs place significant pressure on national healthcare systems, particularly in countries with rapidly aging populations. Studies highlight that investments in preventive exercise programs can substantially reduce fall-related healthcare expenses and improve cost-efficiency in the management of older adult health [17]. Moreover, community-level exercise programs support social participation, psychological well-being, and improved quality of life, extending their benefits beyond physical health alone [22,23].

A wide range of exercise modalities has been studied for their potential to reduce fall risk, each focusing on different physiological mechanisms [24]. Strength training programs aim to counteract age-related muscle loss and improve the ability to generate force during daily activities [25]. Balance training interventions target sensory integration, postural alignment, and anticipatory movement adjustments [26]. Multimodal exercise programs incorporate elements of both strength and balance, alongside flexibility, gait training, and functional mobility exercises, thereby addressing multiple determinants of fall risk simultaneously [6,15]. Additionally, Tai Ji Quan has been extensively examined and shown to enhance proprioception, controlled breathing, dynamic balance, and lower-limb coordination, making it particularly effective for older adults with balance limitations [14].

Although this approach is not included as a core reference in your introduction sources, its conceptual relevance is grounded in decades of evidence demonstrating that improved reactive stepping is a critical component of fall prevention [11]. Reactive balance capability represents one of the strongest predictors of fall risk because most real-world falls occur during unexpected perturbations, such as uneven surfaces, sudden turns, or slips during locomotion [27]. Strengthening the neuromuscular responses required to counteract these perturbations aligns with evidence showing that functional and neuromuscular training significantly enhances fall resilience [28].

Despite well-established evidence supporting exercise programs, older adults often face barriers that impede their participation. These include fear of injury, low motivation, limited access to exercise facilities, and the misconception that age restricts the capacity to benefit from physical training [7]. However, research consistently shows that even low- to moderate-intensity exercise can lead to meaningful improvements in physical performance among older adults. Furthermore, community-delivered programs such as Tai Ji Quan classes, supervised balance groups, and home-based strength programs offer scalable, affordable, and culturally adaptable approaches to promoting active aging. These programs provide a structured environment for older adults to engage in safe and effective exercise, with several studies emphasizing their high adherence rates and positive psychological effects [10,14].

Alongside physical benefits, exercise participation contributes to enhanced cognitive and psychological well-being in older adults, supporting a more holistic model of fall prevention. This aligns with contemporary perspectives in physical education and sports science, which view movement not only as a means to enhance physical capacity but also as a transformative experience that shapes mental resilience and promotes emotional stability [10]. Integrating mental and physical benefits of exercise strengthens the rationale for adopting exercise-based approaches as comprehensive fall-prevention strategies.

The compelling evidence supporting exercise interventions has shaped global public health recommendations. Leading organizations, including the World Health Organization, advocate for structured exercise programs emphasizing balance, strength, and mobility training as core components of fall-prevention strategies for community-dwelling older adults [2]. These guidelines stress that such programs should be accessible, evidence-based, and delivered in a manner that considers individual health status, cultural context, and personal preferences. By adopting these recommendations, healthcare systems can proactively address fall risk, reduce long-term healthcare expenditures, and enhance functional independence among older adults.

Despite the growing body of literature supporting exercise-based fall prevention, the current evidence base remains fragmented. Most previous systematic reviews have examined single exercise modalities in isolation, such as strength training, balance training, Tai Ji Quan, or perturbation-based approaches, without providing a unified comparison across these distinct intervention types. As a result, clinicians and policymakers lack an integrated synthesis that contrasts traditional balance- and strength-based programs with emerging reactive balance and perturbation-based training within a single evidence framework. Moreover, the mechanisms through which different exercise modalities reduce fall risk, such as improvements in anticipatory balance, reactive stepping, neuromuscular strength, and long-term adherence, have rarely been examined together. Therefore, this systematic review uniquely addresses this gap by comparatively synthesizing randomized controlled trials investigating Tai Ji Quan, the Otago Exercise Program, multimodal strength–balance training, and perturbation-based reactive balance interventions in community-dwelling older adults, to clarify their relative effectiveness, mechanisms of action, and practical implications for fall prevention. The review was structured according to a PICO framework, comparing different exercise modalities (I) against usual care or alternative exercise (C) in community-dwelling older adults (P), with fall incidence, balance, mobility, and strength as primary outcomes (O).

## 2. Materials and Methods

### 2.1. Study Selection Procedures

The review protocol was not preregistered due to the exploratory and integrative nature of the review, which aimed to synthesize diverse and emerging exercise modalities rather than evaluate a narrowly defined intervention. The study selection process followed the PRISMA 2020 Figure 1 (Preferred Reporting Items for Systematic Reviews and Meta-Analyses) and PROSPERO guidelines [29] and involved multiple stages to ensure systematic identification and inclusion of relevant studies. The review protocol was not preregistered before conducting the study. The research included studies conducted from 2010 to 2025, covering the last 15 years and ensuring up-to-date findings that reflect current trends in the field. The search was conducted until 15 August 2025, with a final update in September 2025. Across the 2010–2025 period, more than half of the included studies (≈52%) were published between 2015 and 2019, reflecting a surge of high-quality research during this phase. In contrast, early publications from 2010–2014 accounted for only about 7% of the studies, while the remaining 41% were produced between 2020 and 2025, highlighting continued contemporary contributions. The studies were searched through the following electronic databases: PubMed, Scopus, Web of Science, and CINAHL. After completing the initial database search, all retrieved records were imported into reference management software, and duplicate entries were removed. Two independent reviewers (SC and PKC) screened titles and abstracts using predefined eligibility criteria, focusing on exercise-based balance, strength, mobility, and reactive-balance interventions for community-dwelling older adults aged 60 years and above. Studies meeting preliminary criteria underwent full-text screening, where the same reviewers independently assessed methodological relevance, intervention focus, and outcome reporting. Conflicts at any stage were resolved through discussion or consultation with a third reviewer (BK). Only studies that fulfilled all inclusion criteria Table 1 and adhered to intervention relevance for fall prevention were included in the final synthesis.

### 2.2. Literature Search: Administration and Update

The literature search was conducted systematically across major scholarly databases including PubMed, Scopus, Web of Science, and CINAHL, using a combination of controlled vocabulary and text-based keywords such as “older adults,” “falls,” “balance training,” “strength training,” “Tai Ji Quan,” “Otago Exercise Program,” “perturbation-based balance training,” and “fall prevention exercises.” Boolean operators were applied to refine retrieval, and filters were limited to English-language, peer-reviewed, full-text publications. The search strategy was designed iteratively, and database-specific adjustments were made to maximize sensitivity. To maintain currency, the search was updated before final analysis to capture newly published randomized or controlled trials meeting eligibility criteria, ensuring the dataset reflected the most recent evidence in fall-prevention exercise interventions.

### 2.3. Data Extraction

Data extraction was performed using a predesigned standardized data extraction protocol [30] to maintain methodological consistency. Extracted information included study identifiers (author, year, country), participant characteristics (sample size, age, eligibility), intervention details (type, frequency, intensity, duration, supervision), comparator groups, outcome variables, measurement instruments, and key findings related to fall incidence, balance performance, mobility, strength, and secondary outcomes. Two independent reviewers conducted the extraction process and cross-checked all entries. Discrepancies were addressed through discussion, and final data were validated before synthesis. When necessary, authors of included trials were contacted for missing or unclear information. When outcome data were missing or unclear, corresponding authors were contacted via email; responses were obtained for a subset of studies, while non-responses were documented and managed conservatively in the synthesis.

### 2.4. Methodological Quality of the Included Studies

The methodological quality of all included randomized and controlled trials was assessed using the Cochrane Risk of Bias 2 (ROB-2) tool. This assessment evaluated potential sources of bias across five domains: (1) the randomization process, (2) deviations from intended interventions, (3) missing outcome data, (4) measurement of outcomes, and (5) selection of reported results, in accordance with the guidance provided in the Cochrane Handbook for Systematic Reviews of Interventions [31]. Each domain was judged as having “low risk,” “some concerns,” or “high risk” of bias, leading to an overall risk-of-bias judgment for each study based on established criteria [32]. Assessments were conducted independently by two reviewers, with disagreements resolved through discussion and consensus, consistent with recommended best practices for minimizing assessor subjectivity [33]. The ROB-2 evaluations were used to inform confidence in the interpretation of outcomes and to guide the narrative weighting of evidence within the synthesis.

### 2.5. Summary Measures

Primary summary measures included fall incidence and injurious falls reported as count data, rate ratios, or risk ratios. Continuous outcomes, such as balance scores (BBS, SPPB), mobility performance (TUG, gait speed), and strength measures (Chair Stand Test, dynamometry), were summarized through means, standard deviations, and mean differences where available. Reactive-balance outcomes were extracted as step-recovery responses or lab-induced fall rates. When multiple measures were reported for the same domain, those most widely validated and consistently applied across trials were prioritized to enhance comparability across studies.

### 2.6. Synthesis of Results

Due to heterogeneity in intervention type, duration, participant characteristics, and outcome assessment methods, a narrative synthesis approach was adopted. Studies were grouped based on intervention modality (e.g., Tai Ji Quan, Otago, multimodal strength-balance training, perturbation-based training), and findings were compared qualitatively across domains of falls, balance, mobility, and strength. Effect direction and consistency were emphasized, and detailed tables were developed to summarize intervention characteristics and outcome results. Where multiple studies reported similar outcomes using comparable tools, patterns of improvement or neutrality were identified to derive cross-study interpretive trends relevant to fall prevention in older adults. Studies reporting laboratory-based biomechanical outcomes, such as slip- or trip-induced balance perturbations and reactive stepping responses, were included alongside community-based fall studies because reactive balance capacity represents a critical mechanistic determinant of fall prevention. Laboratory-based outcomes were analyzed and interpreted separately from community fall incidence, with a clear distinction maintained between mechanistic adaptations (reactive balance and compensatory stepping) and clinical endpoints (actual fall occurrence). This approach allowed biomechanical insights derived from controlled laboratory settings to be contextualized without overextending causal inference to real-world fall reduction.

### 2.7. Data Synthesis

A quantitative meta-analysis was not conducted due to substantial clinical and methodological heterogeneity across included trials, including differences in intervention modalities, outcome definitions, follow-up durations, and participant risk profiles. Formal statistical heterogeneity indices (e.g., I^2^ or τ^2^) were therefore not calculated, as pooling effect estimates under these conditions would risk producing misleading summary effects. Instead, a structured narrative synthesis was undertaken, emphasizing consistency in the direction of effects across studies rather than pooled magnitude estimates. Consistent with PRISMA 2020 guidance, the certainty of evidence was interpreted qualitatively, and a formal GRADE assessment was not applied because no quantitative synthesis was performed.

### 2.8. Publication Bias

Publication bias was considered by examining the completeness of available data, selective reporting tendencies identified during the risk-of-bias assessment, and the distribution of study outcomes across different intervention modalities. Although formal statistical tests for publication bias (e.g., funnel plots or Egger’s regression) were not feasible due to substantial heterogeneity in measurement scales, intervention types, and study designs, this approach is consistent with PRISMA recommendations for narrative syntheses when meta-analytic pooling is inappropriate [29]. Qualitative assessment methods, supported by guidance from the Cochrane Handbook, allowed for the evaluation of selective reporting patterns and missing data across included trials [31]. Overall, most included studies were published in peer-reviewed journals with transparent reporting practices, reducing but not eliminating the potential for selective publication favoring statistically significant or positive findings, as noted by [32].

### 2.9. Additional Analyses

Additional analyses included subgroup comparisons based on intervention type (balance-focused, strength-focused, multimodal, perturbation-based), duration (short-term ≤ 12 weeks vs. long-term ≥ 6 months), and delivery mode (home-based vs. supervised group programs). Trends were also evaluated by age range and fall-risk profile to determine differential responsiveness to specific intervention strategies. Sensitivity considerations were applied by giving greater interpretive weight to low-risk-of-bias RCTs while acknowledging outcomes from smaller pilot trials and studies with methodological limitations. These additional analyses supported a more nuanced understanding of which exercise interventions were most effective for reducing falls and improving physical functioning in older adults.

## 3. Results

A total of 27 studies Table 2 met the eligibility criteria and were included in the final synthesis. These studies varied in intervention type, duration, sample size, and outcome measures, providing a diverse representation of exercise-based strategies for fall prevention among community-dwelling older adults. The selection process is summarized in the PRISMA flow diagram and reflects rigorous screening across multiple databases, removal of duplicates, and detailed full-text evaluation. The included studies comprised randomized controlled trials, cluster RCTs, controlled clinical trials, and pilot interventions, with sample sizes ranging from small feasibility trials to large multi-center investigations. The following subsections present the synthesized results organized by primary and secondary outcome domains, highlighting patterns of effectiveness and variability across intervention types.

Table 3 summarizes the methodological quality of all included trials using the Cochrane ROB-2 tool, illustrating variations in risk of bias across the five assessment domains. Most studies demonstrated low risk in randomization, adherence to interventions, and outcome measurement, although several exhibited “some concerns” due to incomplete follow-up or selective reporting.

Pilot studies and cluster designs showed slightly higher levels of uncertainty because of small samples, attrition, or limited allocation detail. This table provides a comprehensive evaluation of internal validity across trials and highlights the relative robustness of the evidence base.

Table 4 presents an overview of the intervention characteristics across all included studies, detailing exercise type, frequency, intensity, session duration, total program duration, and delivery format. The interventions varied considerably, ranging from long-term community-based strength-balance programs to short-term perturbation-based reactive balance training.

### 3.1. Long-Term Adherence and Sustainability

This variation reflects the diversity of fall-prevention strategies employed in older adult populations. The table provides a clear comparison of intervention structures, enabling an understanding of their scope, intensity, and practical applicability.

Table 5 summarizes the effects of the included interventions on fall incidence and injurious falls, demonstrating that most exercise programs resulted in meaningful reductions in fall frequency compared with control or usual-care groups. The strongest effects were observed in Tai Ji Quan, Otago, multimodal strength-balance programs, and perturbation-based training.

### 3.2. Reactive Balance Improvement

These findings highlight the consistent protective impact of exercise-based interventions on fall risk. The table provides a concise comparison of outcome direction and study-specific observations.

Table 6 outlines the improvements in balance performance across interventions using validated instruments such as the Berg Balance Scale, Timed Up and Go, and post-urography. Most studies reported positive effects, with notable improvements following Tai Ji Quan, Otago, multimodal training, and perturbation-based programs. These findings underscore the importance of targeted balance exercises in enhancing postural control and reactive balance in older adults. The table helps visualize the consistency of balance-related benefits across diverse exercise modalities.

Table 7 provides a summary of mobility and functional performance outcomes, demonstrating that exercise interventions frequently improved gait speed, TUG performance, and SPPB scores. Programs involving strength-balance combinations, balance circuits, and perturbation training showed particularly strong impacts on functional mobility.

### 3.3. Strength and Mobility Adaptations

These improvements reflect enhanced physical functioning and reduced fall risk among participants. The table highlights consistent positive effects across a wide range of functional mobility assessments.

Table 8 details the effects of various interventions on lower-limb strength, showing consistent improvements across group exercise programs, balance circuits, multimodal training, and strength-balance combinations. Strength gains were commonly measured using chair stand tests, dynamometry, and force platforms, demonstrating enhanced muscular performance and power.

These findings emphasize the important contribution of strength development to overall fall-prevention effectiveness. The table provides clear evidence of the widespread benefits of strength-oriented interventions.

## 4. Discussion

The present systematic review synthesized evidence from 27 clinical trials investigating the effects of balance-, strength-, mobility-, and perturbation-based exercise interventions on fall incidence, balance performance, mobility, and lower-limb strength in community-dwelling older adults. Across studies, a consistent pattern emerged demonstrating that structured exercise programs, particularly those emphasizing balance control, reactive stability, and lower-limb strength, produce meaningful improvements in physical function and reduce the risk of falls among older populations. The convergence of evidence from diverse methodologies, ranging from long-term community trials to short-term laboratory-based perturbation training, provides a robust foundation for understanding the multifaceted role of exercise in fall prevention.

The included studies demonstrated substantial clinical and methodological heterogeneity with respect to intervention duration, training intensity, delivery mode, outcome measures, and participant risk profiles. While this variability precluded quantitative meta-analysis, it reflects real-world diversity in fall-prevention practice. Consequently, conclusions should be interpreted as indicative of consistent directional benefits rather than precise effect magnitudes. Importantly, the convergence of positive outcomes across heterogeneous designs strengthens the external validity of exercise-based fall-prevention strategies, although generalizability to very frail or institutionalized populations remains limited.

Our findings demonstrate a consistent improvement in balance performance across multiple interventions included in this review, particularly in studies employing Tai Ji Quan, perturbation-based balance training, and multimodal strength-balance programs [34,38,42,44,46,55,56,57]. These improvements closely mirror the broader evidence base, where Tai Ji Quan has repeatedly been shown to enhance functional balance, postural stability, and reactive control in older adults. In the present review, Tai Ji Quan interventions not only reduced fall incidence but also produced measurable gains in balance function, consistent with the large clinical trials conducted by [42,46]. Mechanistically, these effects may be attributed to Tai Ji Quan’s emphasis on slow, controlled weight shifting, proprioceptive refinement, and continuous center-of-mass regulation, which directly translate into safer movement strategies during daily activities. Similarly, the strong improvements observed in perturbation-based programs [34,44,47] align with previous research showing that repeated exposure to slips, trips, and destabilizing forces enhances reactive stepping, shortens response latency, and strengthens neuromuscular coordination under unexpected loss-of-balance conditions. Together, these patterns indicate that both anticipatory and reactive balance systems adapt robustly to targeted training, offering a strong mechanistic basis for the observed reductions in fall risk across interventions.

In our review, several studies demonstrated clear improvements in balance, mobility, and fall reduction through the Otago Exercise Program (OEP). Specifically, Arkkukangas et al. [49] reported meaningful gains in balance and adherence, Waters et al. [58] documented significant reductions in fall rates among Native American elders, and Cederbom & Arkkukangas [50] showed sustained functional benefits during long-term follow-up. Collectively, these findings highlight OEP’s consistent effectiveness across different populations and study durations.

These results align closely with previous evidence, which shows that structured exercise reduces falls by approximately 15% (rate ratio: 0.85) across large-scale trials [61,62]. Other research has similarly confirmed that balance-strength programs particularly Otago and Tai Ji Quan, enhance lower-limb strength, joint stability, and postural control [63,64].

When viewed together, both our included studies and existing literature suggest that longer, progressively loaded interventions (≥12 months) are associated with even greater reductions in fall risk, reinforcing the importance of sustained engagement for neuromuscular adaptation. Mechanistically, OEP’s effectiveness stems from its progressive lower-limb strengthening and dynamic balance training, which enhance proprioception and functional stability. These adaptations improve anticipatory postural adjustments and gait control, reducing vulnerability to balance disturbances. Such neuromuscular benefits are consistent with prior evidence showing that structured strength-balance programs enhance sensorimotor function and fall resilience in older adults [25,27]. Recent reviews further confirm that home-based, task-specific balance training improves confidence and reduces fear of falling, contributing to lower fall incidence [23,24].

In this systematic review, several included trials demonstrated consistent and meaningful improvements in reactive balance and fall-recovery ability following perturbation-based balance training. Studies such as Mansfield et al. [34], Okubo et al. [47], Allin et al. [51], Aviles et al. [44], and Nørgaard et al. [59] showed that repeated exposure to slips, trips, or rapid postural challenges produced substantial gains in compensatory stepping, reactive stability, and reductions in laboratory-induced falls. Importantly, these benefits emerged even during relatively short intervention periods (4–8 weeks), highlighting the efficiency of perturbation training in eliciting rapid neuromuscular adaptation in older adults. These findings align strongly with previous research demonstrating that high-task-specific perturbation exposure can recalibrate automatic postural responses, accelerate neuromuscular activation, and enhance trunk control during unexpected disturbances. For example, Allin et al. [51] reported significant reductions in both slip- and trip-related falls following treadmill-based perturbations, while Aviles et al. [44] observed superior improvements in reactive balance compared to traditional Tai Ji Quan training. Similarly, the more recent RCT by Nørgaard et al. [59] confirmed that structured perturbation protocols translate into measurable reductions in real-world fall rates, reinforcing the clinical applicability of this approach. Even smaller pilot investigations, such as Okubo et al.’s [48] unpredictability training study, demonstrated that brief exposures can induce meaningful motor learning across both younger and older adults. Taken together, our results support the growing evidence that perturbation-based balance training directly targets the fast, automatic protective responses required to prevent falls. By improving reactive stepping, enhancing proprioceptive sensitivity, and promoting more efficient neuromuscular control, these programs offer a highly potent and time-efficient strategy for fall prevention in community-dwelling older adults.

Beyond falls and reactive balance, many studies reported substantial gains in traditional balance outcomes, such as Berg Balance Scale scores, TUG performance, and posturographic measures. Improvements in these outcomes were observed across diverse interventions, including Tai Ji Quan [42,46], multimodal training [37], Otago [58], balance circuits [56], and strength-balance combinations [38]. These consistent gains suggest that balance-focused exercise induces robust neuromuscular adaptations regardless of specific training modality. Many of these improvements were accompanied by enhancements in mobility, particularly in gait speed, TUG, and SPPB scores. In a study by Liu-Ambrose et al. [45] demonstrated that a home-based strength-balance program yielded meaningful mobility gains alongside reductions in fall incidence, highlighting the interconnectedness of balance and functional mobility in determining overall fall risk.

Another key theme across studies was the improvement of lower-limb muscular strength, which emerged as a crucial mediator of enhanced mobility and balance. LaStayo et al. [52] reported that eccentric resistance training produced greater improvements in lower-limb strength and functional performance than traditional resistance exercise, with no significant differences in fall outcomes. This suggests that eccentric loading enhances neuromuscular and functional capacity, which may indirectly support balance and mobility. Kulkarni et al. [57], Costa et al. [56], and Lacroix et al. [38] all reported significant increases in muscle strength or power following structured exercise interventions. These gains are particularly important given the established relationship between lower-limb weakness and fall risk in older adults. Strength improvements also contributed to better performance on functional tests such as the chair stand and SPPB, demonstrating meaningful carryover from isolated strength adaptations to broader mobility gains. Importantly, strength improvements were not limited to traditional resistance training; even balance-oriented or multimodal programs such as Otago and Stay Balanced produced gains in lower-limb strength, likely due to their emphasis on functional, weight-bearing exercises. Gianoudis et al. [54] showed that a long-term multimodal program with high-velocity resistance and balance training significantly improved strength, power, dynamic balance, and bone density in older adults. However, the absence of a significant reduction in fall incidence underscores the distinction between musculoskeletal gains and direct fall outcomes, highlighting the need for task-specific balance challenges when fall prevention is the primary goal. Importantly, improvements in biomechanical, neuromuscular, or laboratory-based outcomes should be interpreted as indicators of enhanced fall resilience rather than direct proxies for reductions in real-world fall incidence.

Some studies introduced alternative models for fall prevention. The STRIDE trial Bhasin et al. [53] implemented a multifactorial, individualized fall-prevention strategy and found reductions in serious fall injuries through coordinated care, risk assessment, and targeted exercise components. Although not exclusively exercise-based, this trial underscored the importance of integrating exercise within broader fall-prevention frameworks. The ProAct65+ trials Stevens et al. [35]; Gawler et al. [39] highlighted the challenges and benefits of implementing community-based strength-balance programs within primary care settings, demonstrating feasibility and improvements in functional outcomes despite variations in implementation fidelity.

An important observation across studies is that program duration significantly influenced outcomes. Long-term interventions (12–24 months), such as those in Patil et al. [37] and El-Khoury et al. [36], produced sustained reductions in falls and injuries, while shorter interventions (4–12 weeks) tended to yield rapid gains in reactive balance and specific neuromuscular outcomes, as demonstrated by [34,47,60]. This diversity in timelines suggests that fall prevention can be achieved through both long-term conditioning and short-term targeted training, depending on specific outcomes of interest. Long-term multimodal and Otago programs appear particularly effective in reducing fall incidence, while shorter perturbation-based interventions may rapidly enhance reactive balance mechanisms. Subgroup analyses indicate that high-risk populations benefit more from these interventions, with a risk ratio of 0.72 for fall rates [61]. Women show a more pronounced benefit from exercise interventions, particularly in fracture prevention, with a risk ratio of 0.37 [64]. In contrast, while exercise interventions are effective, some studies suggest that multifactorial approaches, which include environmental modifications and education, may offer additional benefits in reducing falls and injuries [62]. This indicates a potential need for integrated strategies in fall prevention. Therefore, Regular participation in physical activity is essential for supporting long-term health, as it helps counteract chronic disease risk, strengthens the musculoskeletal system, improves balance control, and ultimately lowers the probability of experiencing falls among older adults [65,66,67].

This systematic review showed that perturbation-based balance training was one of the most consistently effective strategies for improving reactive stepping, compensatory balance, and laboratory-induced fall recovery. Studies by [47], Allin et al. [51], Aviles et al. [44], and Mansfield et al. [34] demonstrated rapid improvements in reactive balance, even in short interventions lasting 4–8 weeks. These findings are further strengthened by Nørgaard et al. [59], who reported significant real-world fall reductions following treadmill-based perturbation training in older adults. The mechanisms observed in our studies are strongly supported by foundational research showing the importance of “change-in-support” strategies [21] and the role of rapid limb movements in balance recovery [28]. Additionally, systematic reviews emphasize that perturbation-based interventions induce unique neural adaptations that traditional balance training does not elicit [27]. Even short-term pilot studies in our review, Okubo et al. [48], demonstrated improvements in motor learning and response speed, aligning with evidence that repetitive exposure to controlled slips and trips recalibrates neuromuscular timing and trunk control strategies. Together, these results indicate that perturbation-based training offers strong mechanistic and clinical value for fall prevention. Across the studies included in our review, the Otago Exercise Program (OEP) consistently improved balance, strength, and mobility, contributing to meaningful reductions in fall risk. For instance, Arkkukangas et al. [49] and Waters et al. [58] demonstrated significant improvements in functional performance and reductions in fall rates, while long-term effects were observed in the follow-up study by [50]. These findings align with broader evidence supporting the effectiveness of progressive strength-balance training in older adults, including recent analyses by Thomas et al. [61] and Guirguis-Blake et al. [62], which reported significant reductions in fall rates with exercise-based interventions. Mechanistically, these improvements are consistent with established physiological principles: targeted lower-limb resistance training enhances neuromuscular force generation [25], improves stability thresholds, and strengthens compensatory mechanisms required for safe ambulation. The OEP’s home-based format also resonates with evidence from community-based program reviews [22,23], emphasizing accessibility and sustainability as key facilitators of long-term adherence. Thus, our results reinforce OEP as a practical and effective intervention for fall prevention. However, while improvements in reactive balance and laboratory-induced fall recovery are indicative of enhanced neuromuscular preparedness, direct translation to reduced real-world fall incidence should be interpreted cautiously, as relatively few trials have examined long-term community fall outcomes following perturbation-based interventions.

Across all included studies, the overall pattern of findings reflects a compelling body of evidence indicating that exercise, irrespective of specific modality, consistently enhances essential physical attributes linked to fall prevention. Whether through traditional balance exercises, strength conditioning, Tai Ji Quan, or reactive perturbation exposure, older adults demonstrated measurable improvements in functional performance, neuromuscular efficiency, postural control, and fall resilience. The convergence of findings from diverse interventions underscores the adaptability of older adults to structured physical training and confirms that targeted exercise remains a central, evidence-based strategy for reducing fall risk.

### 4.1. Implications of the Study

The findings of this systematic review hold important implications for clinical practice, community-based health programs, and public health policy. The consistent effectiveness of balance-, strength-, and perturbation-based exercise interventions demonstrates that structured physical training is a powerful and modifiable strategy for reducing fall risk in older adults. Evidence from Tai Ji Quan, Otago, and multimodal programs suggests that exercise can be safely implemented across diverse settings, including home-based programs, community centers, primary care environments, and specialized clinical facilities. These results reinforce the need for integrating exercise prescriptions into routine geriatric care and fall-prevention protocols, emphasizing that even low-intensity, accessible interventions can produce clinically meaningful improvements. The growing evidence supporting perturbation-based training highlights new opportunities for innovation in fall-prevention approaches that target reactive balance mechanisms. Overall, these implications underscore the necessity of prioritizing structured exercise interventions as a foundational element of fall-prevention strategies at individual, clinical, and public health levels. Accordingly, percentage reductions in falls are reported descriptively to illustrate directional trends rather than inferential estimates, and should not be interpreted as pooled or adjusted effect sizes.

### 4.2. Study Limitations

This review has several limitations that should be acknowledged. First, substantial clinical and methodological heterogeneity existed across studies with respect to intervention type, duration, intensity, outcome measures, and follow-up periods, which limited the feasibility of a pooled meta-analysis and necessitated a narrative synthesis approach. Also, substantial variability existed in the biomechanical and functional assessment tools used across the included studies, including force platforms, wearable sensors, clinical balance scales, and laboratory-based perturbation systems. The lack of standardization in balance and reactive response measurements limits direct comparability across trials and may influence the interpretation of mechanistic findings relative to clinical outcomes.

Second, outcome measures and reporting formats varied widely, with some trials relying on self-reported falls or adherence, potentially introducing measurement bias. Third, several included studies were small pilot or feasibility trials with limited sample sizes, which may reduce the generalizability of findings. Fourth, although comprehensive search strategies were employed, only English-language, peer-reviewed full-text articles were included, which may increase the risk of language or publication bias. Finally, incomplete reporting of allocation concealment, blinding, or long-term follow-up in some studies contributed to “some concerns” ratings in the risk-of-bias assessment and may influence confidence in the strength of the evidence. Accordingly, the findings of this review should be interpreted as evidence of consistent directional benefits of exercise-based interventions rather than definitive comparative or causal conclusions.

### 4.3. Recommendations for Future Research and Practice

Future research should prioritize the standardization of outcome measures, particularly fall incidence, reactive balance, mobility tests, and lower-limb strength assessments, to enable more robust comparisons and meta-analytic synthesis. Long-term follow-up studies are needed to better understand the sustainability of intervention effects, particularly for perturbation-based and multimodal programs. Researchers should also explore the integration of technology-assisted balance training, such as sensor-based feedback and virtual perturbation systems, which may enhance training specificity and adherence. Greater emphasis should be placed on recruiting more diverse populations, including those over 80 years, individuals with chronic conditions, and underrepresented cultural groups. Clinically, practitioners are encouraged to incorporate structured balance and strength training into routine geriatric care, adapt interventions based on individual risk profiles, and promote adherence through behavioral support strategies. Finally, combining traditional balance and strength programs with perturbation-based training may offer a more comprehensive approach to fall prevention and should be explored in future randomized trials.

## 5. Conclusions

This systematic review demonstrates that exercise-based interventions targeting balance, lower-limb strength, and reactive postural control are effective in reducing fall risk and improving functional performance in community-dwelling older adults. Strong and consistent evidence supports Tai Ji Quan, the Otago Exercise Program, and multimodal strength–balance training for reducing real-world falls, while perturbation-based balance training shows robust mechanistic benefits by enhancing reactive stepping and fall-recovery responses. From a practical perspective, fall-prevention programs should prioritize structured, progressive balance-strength exercises and, where feasible, incorporate task-specific reactive balance training to address unexpected perturbations encountered in daily life. Future research should focus on standardized outcome measures, longer follow-up periods, and scalable community-based delivery models to strengthen evidence translation into routine geriatric care and public health policy.

## Figures and Tables

**Figure 1:**
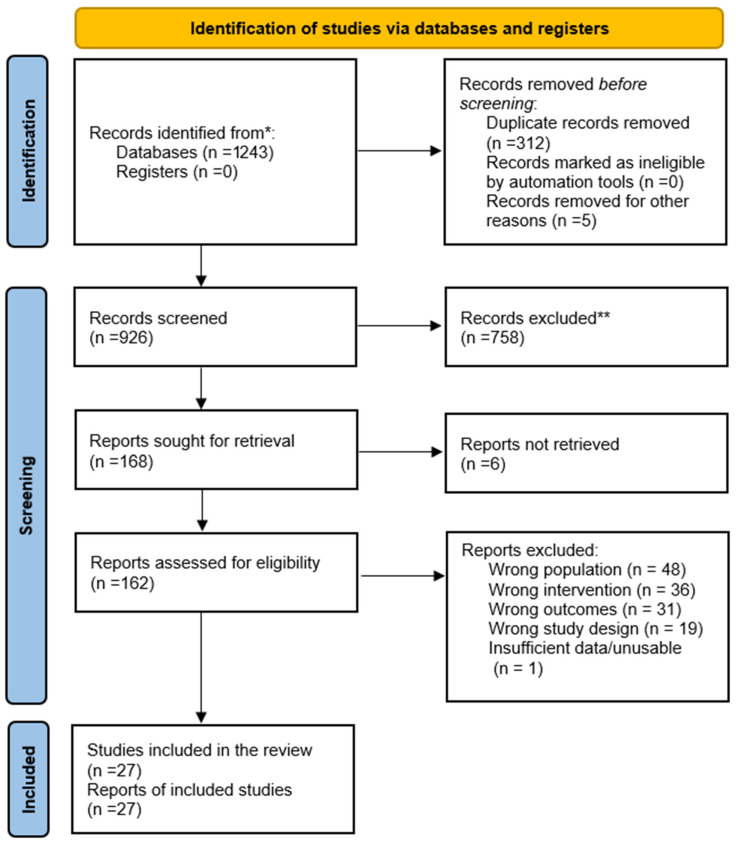
PRISMA 2020 flow diagram showing the study selection process for the systematic review. * the number of records identified from each database or register, ** indicate how many records were excluded.

**Table 1:** Inclusion and Exclusion Criteria.

Criteria Category	Inclusion Criteria	Exclusion Criteria
Population	Community-dwelling older adults aged ≥60 years.	Adults < 60 years.
	Individuals with a history of falls, balance impairment, or mobility limitation.	Individuals with conditions preventing exercise participation (severe dementia, severe stroke, late-stage neurological disease).
	Both male and female participants living independently in the community.	Residents of long-term institutional care or nursing homes.
Intervention/Exposure	Exercise-based interventions focused on balance, strength, mobility, or reactive balance.	Non-exercise interventions (education-only, medication-only, surgery).
	Programs such as: Tai Ji Quan, Otago, perturbation-based balance training, multimodal strength–balance programs. Exercise-based interventions focused on balance, strength, mobility, or reactive balance with a minimum duration of ≥4 weeks.	Aerobic-only programs with no balance or strength component.
	Home-based, community-based, supervised, or laboratory-based exercise interventions.	Technology-only tools with no active physical exercise component.
Primary Outcomes	Fall incidence during or after intervention.	Studies not reporting fall-related outcomes.
	Injurious falls.	Studies reporting only subjective perceptions without physical outcomes.
	Measures of balance or mobility.	Outcomes irrelevant to fall prevention or physical performance.
Secondary Outcomes	Lower-limb strength.	Studies reporting only psychosocial, emotional, or qualitative data.
	Reactive balance responses to slips/trips.	Studies with no physical or functional measurement.
	Fear of falling, quality of life, pain.	Cognitive-only or questionnaire-only studies unrelated to fall mechanics.
Study Designs	Randomized controlled trials (RCTs).	Editorials, commentaries, letters, opinion papers.
	Cluster RCTs, controlled trials, pilot RCTs.	Systematic reviews, meta-analyses (not eligible for extraction).
	Prospective follow-up trials linked to an intervention.	Protocol papers without outcome data.
		Cross-sectional, retrospective, or case–control studies.
Language	Full-text studies published in English.	Non-English studies without translation.
Publication Type	Peer-reviewed journal articles with complete methodology.	Theses, dissertations, posters, book chapters, unpublished works.
Time Frame	No restriction on publication year.	
Accessibility	Full-text available and retrievable.	Full-text not accessible after reasonable attempts.

**Table 2:** Study Characteristics.

Author & Year	Country	Study Design	Participants (*N*, Age)	Intervention	Comparison	Duration	Outcome Measures	Instruments	Key Findings
Mansfield et al., 2010 [34]	Canada	RCT	Total *N* = 30 (Intervention group, *n* = 15; Control group, *n* = 15), ≥65 years	Perturbation stepping/grasping	Flexibility training	6 weeks	Compensatory stepping	Force plates, motion analysis	Improved stepping/grasping fall-recovery reactions
Stevens et al., 2013 [35]	UK	Cluster RCT	Total *N* = 1256 (Intervention and control clusters), ≥65 years	ProAct65+ strength + balance	Usual GP care	24 months	Falls, mobility	GP records, BBS	Identified challenges in multi-site exercise implementation
El-Khoury et al., 2015 [36]	France	RCT	*N* = 706, women 75–85 years	2-year balance training	No exercise	24 months	Fall-induced injuries	Physician-verified injury reports	Long-term balance training reduced moderate injuries
Patil et al., 2015 [37]	Finland	RCT	*N* = 409, women 70–80 years	Multimodal training	Usual physical activity	24 months	Falls, physical function	SPPB, fall diaries	Reduced falls and improved mobility
Lacroix et al., 2016 [38]	Switzerland	RCT	*N* = 75, 65–80 years	Supervised vs. unsupervised balance + strength	Unsupervised exercise	12 weeks	Balance, muscle power	Force plates, BBS	Supervised training yielded superior gains
Gawler et al., 2016 [39]	UK	RCT	*N* = 1256, ≥65 years	ProAct65+ exercise program	Usual care	24 weeks	Falls, balance	BBS, TUG, fall diaries	Exercise intervention reduced fall rates
Trombetti, et al. 2016 [40]	Switzerland	RCT	*N* = 134, ≥65 years	Music-based multitask balance + gait training	Usual care	12 weeks	Gait, balance, fall risk	GAITRite, BBS, TUG	Significant improvements in gait variability and balance, reducing fall risk.
Boongird et al., 2017 [41]	Thailand	RCT	*N* = 484, ≥65 years	Home-based strength & balance	Usual care	12 months	Falls, mobility	FES-I, fall logs	Home-based program reduced falls significantly
Li et al., 2018 [42]	USA	RCT	Total *N* = 670 (Intervention group, *n* = 335; Control group, *n* = 335), ≥70 years	Therapeutic Tai Ji Quan	Multimodal exercise	24 weeks	Fall incidence, functional balance	FES-I, TUG, fall logs	Tai Ji Quan significantly reduced falls compared to multimodal exercise
Jansen et al., 2018 [43]	Germany	Randomized non-inferiority trial	*N* = 310, ≥70 years	Group-delivered LiFE (Lifestyle-Integrated Functional Exercise) program	Individually delivered LiFE program	12 weeks intervention + 6-month follow-up	Physical function, balance, falls, activity levels	TUG, SPPB, fall calendars, accelerometers	Group-delivered LiFE was non-inferior to individual LiFE in improving functional ability, balance, and daily activity levels; both formats reduced fall risk.
Aviles et al., 2019 [44]	USA	Pilot trial	*N* = 48, ≥65 years	Trip-like treadmill	Tai Ji Quan	8 weeks	Reactive balance	Motion analysis	Trip-like training is superior for reactive stability
Liu-Ambrose et al., 2019 [45]	Canada	RCT	*N* = 344, ≥70 years	Home-based strength & balance program	Usual care	12 months	Falls, mobility, balance	Fall calendars, SPPB, gait tests	Reduced subsequent falls and improved mobility in high-risk older adults
Li et al., 2019 [46]	USA	RCT follow-up	*N* = 670, ≥70 years	Tai Ji Quan	Multimodal & stretching	6 months follow-up	Injurious falls	Clinical fall surveillance	Tai Ji Quan reduced injurious falls over extended follow-up
Okubo et al., 2019 [47]	Australia	RCT	*N* = 46, ≥65 years	Reactive balance (slips/trips)	Strengthening exercises	6 weeks	Balance recovery	Slip/trip treadmill system	Improved reactive balance and reduced lab-induced falls
Okubo et al., 2019 [48]	Australia	Pilot RCT	*N* = 40, young & older adults	Slip/trip unpredictability training	Basic reactive training	4 weeks	Biomechanical responses	Vicon motion analysis	Improved balance recovery under unpredictable conditions
Arkkukangas et al., 2019 [49]	Sweden	RCT	*N* = 175, 65–80 years	Otago + behavior change support	Otago alone	3 months	Balance, adherence	BBS, adherence logs	Behavior support improved adherence but did not significantly alter fall outcomes
Cederbom & Arkkukangas, 2019 [50]	Sweden	Follow-up study	*N* = 230, ≥65 years	Otago program		3 years	Pain, function	Self-reported pain scales	OEP reduced long-term musculoskeletal pain
Allin et al., 2020 [51]	USA	RCT	*N* = 161, ≥65 years	Perturbation-based balance	Flexibility training	6 weeks	Falls (slip & trip)	Slip-trip treadmill	Reduced both slip- and trip-related falls
LaStayo et al., 2017 [52]	USA	Randomized controlled trial	*N* = 64, >65 years)	Eccentric resistance exercise within a multicomponent fall-reduction program	Traditional resistance exercise	12 weeks	Falls, balance, strength, mobility	Fall reports, functional mobility tests	Greater functional gains; similar fall outcomes
Bhasin et al., 2020 [53]	USA	RCT	*N* = 5451, ≥70 years	Multi-factorial intervention + fall risk assessment	Enhanced usual care	3 years	Serious fall injuries	Medical documentation	Multifactorial intervention produced modest improvement
Gianoudis et al., 2014 [54]	Australia	Randomized controlled trial	Total *N* = 162 (Intervention group, *n* = 81; Control group, *n* = 81),	Targeted multimodal exercise program combining high-velocity resistance training, weight-bearing impact, and balance exercises.	Usual activity (control)	12 Months	Falls (secondary outcome), balance, muscle strength, functional muscle power, bone mineral density	Fall calendars, Timed Stair Climb Test, Four Square Step Test, Sit-to-Stand, dual-energy X-ray absorptiometry (DXA)	Significant improvements in muscle strength, functional power, balance, and bone mineral density, with no significant reduction in fall incidence compared with controls.
Halvarsson et al., 2021 [55]	Sweden	Cluster RCT	*N* = 573, ≥70 years	StayBalanced balance program	Usual care	6 months	Falls, balance	BBS, fall diaries	Effective implementation of evidence-based training
Costa et al., 2022 [56]	Brazil	RCT crossover	*N* = 28, ≥65 years	Balance exercise circuit	Traditional exercise	12 weeks	Postural control	Force platforms	Circuit improved static & dynamic balance
Kulkarni et al., 2022 [57]	India	RCT	*N* = 120, ≥60 years	Group balance + strength program	No exercise	12 weeks	Fall risk, strength	BBS, MMT	Significant improvements in balance & fall-related fear
Waters et al., 2022 [58]	USA (Zuni tribe)	RCT	*N* = 121, 65–95 years	Otago program	Fall prevention education	6 months	Falls, balance	TUG, fall diaries	Otago reduced fall risk in Native American elders
Nørgaard et al., 2023 [59]	Denmark	RCT	*N* = 140, 65–90 years	Treadmill perturbation	No training	3 months	Fall rates	Wearable sensors, fall reports	Significant reduction in laboratory-induced falls
Petrovic et al., 2024 [60]	Germany	RCT	*N* = 60, ≥70 years	Perturbation treadmill	Usual physiotherapy	4 weeks	Mobility, balance	SPPB, gait speed	Improved balance and gait in geriatric patients

Notes: RCT = Randomized Controlled Trial; cRCT = Cluster Randomized Controlled Trial; PBRT = Perturbation-Based Balance Training; OEP = Otago Exercise Program; MME = Multimodal Exercise; TTT = Treadmill Trip-Like Training; TJQ = Tai Ji Quan; SPPB = Short Physical Performance Battery; TUG = Timed Up and Go Test; BBS = Berg Balance Scale; FES-I = Falls Efficacy Scale-International; IMU = Inertial Measurement Unit; QoL = Quality of Life; ADL = Activities of Daily Living; HRQoL = Health-Related Quality of Life; GP = General Practice; MoCA = Montreal Cognitive Assessment; MMSE = Mini-Mental State Examination; RT = Reactive Training; MT = Multimodal Training; PT = Physiotherapy; BMI = Body Mass Index.

**Table 3:** Risk of Bias (ROB-2) for Included Studies.

Study (Author, Year)	Randomization Process	Deviations from Intended Interventions	Missing Outcome Data	Measurement of the Outcome	Selection of the Reported Result	Overall Risk of Bias
Mansfield et al., 2010 [34]	Some concerns	Low	Low	Low	Some concerns	Some concerns
Stevens et al., 2013 [35]	Some concerns	Low	Some concerns	Some concerns	Some concerns	Some concerns
El-Khoury et al., 2015 [36]	Low	Low	Some concerns	Low	Some concerns	Some concerns
Patil et al., 2015 [37]	Low	Low	Some concerns	Low	Some concerns	Some concerns
Lacroix et al., 2016 [38]	Low	Low	Low	Low	Some concerns	Low
Gawler et al., 2016 [39]	Some concerns	Low	Some concerns	Low	Some concerns	Some concerns
Boongird et al., 2017 [41]	Some concerns	Low	Some concerns	Low	Some concerns	Some concerns
Li et al., 2018 [42]	Low	Low	Low	Low	Some concerns	Low
Jansen et al., 2018 [43]	Low	Low	Low	Low	Some concerns	Low
Aviles et al., 2019 [44]	Some concerns	Low	Some concerns	Low	Some concerns	Some concerns
Liu-Ambrose et al., 2019 [45]	Low	Low	Low	Low	Some concerns	Low
Li et al., 2019 [46]	Low	Low	Low	Low	Some concerns	Low
Okubo et al., 2019 (RCT) [47]	Low	Low	Low	Low	Some concerns	Low
Okubo et al., 2019 (Pilot RCT) [48]	Some concerns	Low	Some concerns	Low	Some concerns	Some concerns
Arkkukangas et al., 2019 [49]	Low	Low	Low	Low	Some concerns	Low
Cederbom & Arkkukangas, 2019 [50]	Some concerns	Low	Some concerns	Some concerns	Some concerns	Some concerns
Allin et al., 2020 [51]	Low	Low	Low	Low	Some concerns	Low
LaStayo et al., 2017 [52]	Low	Low	Some concerns	Low	Some concerns	Some concerns
Bhasin et al., 2020 [53]	Low	Low	Low	Low	Low	Low
Gianoudis et al., 2014 [54]	Low	Low	Low	Low	Some concerns	Low
Halvarsson et al., 2021 [55]	Some concerns	Low	Some concerns	Low	Some concerns	Some concerns
Costa et al., 2022 [56]	Some concerns	Low	Low	Low	Some concerns	Some concerns
Kulkarni et al., 2022 [57]	Some concerns	Low	Some concerns	Some concerns	Some concerns	Some concerns
Waters et al., 2022 [58]	Low	Low	Low	Low	Some concerns	Low
Nørgaard et al., 2023 [59]	Low	Low	Low	Low	Some concerns	Low
Trombetti et al., 2016 [40]	Low	Low	Low	Low	Some concerns	Low
Jansen et al., 2018 [43]	Low	Low	Low	Low	Some concerns	Low
Petrovic et al., 2024 [60]	Low	Low	Low	Low	Some concerns	Low

**Table 4:** Summary of Intervention Characteristics Across Included Studies.

Study (Author, Year)	Intervention Type	Frequency (Sessions/Week)	Intensity	Session Duration	Total Duration	Mode of Delivery/Setting
Mansfield et al., 2010 [34]	Compensatory stepping & grasping	2×/week	High (perturbation-based)	45 min	6 weeks	Lab-based platform
Stevens et al., 2013 [35]	ProAct65+ strength & balance	1×/week + home	Low-moderate	60 min	24 weeks	Community + home
El-Khoury et al., 2015 [36]	Long-term balance training	1–2×/week	Moderate	45–60 min	24 months	Group-based supervised
Patil et al., 2015 [37]	Multimodal exercise	2–3×/week	Moderate	60 min	24 months	Group supervised
Lacroix et al., 2016 [38]	Combined balance + strength	2×/week	Moderate-high	60 min	12 weeks	Supervised vs. unsupervised home program
Gawler et al., 2016 [39]	ProAct65+ exercise	1×/week + home	Low-moderate	60 min	24 weeks	Primary care + home
Trombetti, et al. (2016) [40]	Music-based multitask balance/gait	1–2×/week	Moderate	60 min	12 weeks	Group-based supervised
Boongird et al., 2017 [41]	Home-based strength + balance	3×/week	Low-moderate	30 min	12 months	Home-based
Li et al., 2018 [42]	Tai Ji Quan (therapeutic)	2×/week	Low-moderate	60 min	24 weeks	Instructor-led group classes
Jansen et al., 2018 [43]	LiFE program (group vs. individual)	3×/week	Low-moderate	30 min	12 weeks + 6-month follow-up	Home-based + group coaching
Aviles et al., 2019 [44]	Trip-like treadmill perturbation	2×/week	Moderate-high	30 min	8 weeks	Lab treadmill perturbations
Liu-Ambrose et al., 2019 [45]	Home-based strength + balance	3–5×/week	Moderate	30–40 min	12 months	Home-based with supervision
Li et al., 2019 [46]	Tai Ji Quan (extended)	2×/week	Low-moderate	60 min	24 weeks + 6 months	Group classes
Okubo et al., 2019 (RCT) [47]	Reactive slip-trip balance	1–2×/week	High (task-specific)	30 min	6 weeks	Lab-based treadmill
Okubo et al., 2019 (Pilot RCT) [48]	Unpredictable slip-trip training	1–2×/week	High	20–30 min	4 weeks	Lab-based
Arkkukangas et al., 2019 [49]	Otago + behavior support	3×/week	Low-moderate	30 min	3 months	Home-based + counseling
Cederbom & Arkkukangas, 2019 [50]	Otago (follow-up)	3×/week	Low-moderate	30 min	12 months	Home-based
Allin et al., 2020 [51]	Slip-trip perturbation	1–2×/week	High	20–30 min	6 weeks	Treadmill-based
Bhasin et al., 2020 [53]	Multifactorial fall-prevention	Variable	Tailored	Variable	3 years	Clinic + home + physiotherapy
Gianoudis et al., 2014 [54]	Multimodal strength–balance–power (Osteo-cise)	3×/week	Moderate–high	60 min	12 months	Community fitness centers
LaStayo et al., 2017 [52]	Eccentric resistance training	3×/week	Moderate–high	45 min	12 weeks	Supervised community-based
Halvarsson et al., 2021 [55]	StayBalanced program	2×/week	Moderate	60 min	12 weeks	Group supervised
Costa et al., 2022 [56]	Balance exercise circuit	3×/week	Moderate	45 min	12 weeks	Group circuit training
Kulkarni et al., 2022 [57]	Group strength-balance	3×/week	Moderate	60 min	12 weeks	Group class
Waters et al., 2022 [58]	Otago Exercise Program	3×/week	Low-moderate	30 min	6 months	Home-based + support
Nørgaard et al., 2023 [59]	Treadmill perturbation	1×/week	High	20–30 min	8 weeks	Lab supervised
Petrovic et al., 2024 [60]	Reactive treadmill perturbations	2×/week	High	30 min	4 weeks	Clinical physiotherapy

**Table 5:** Outcome Summary for Fall Incidence.

Study	Intervention	Comparison	Outcome Measure	Effect Direction	Notes
Liu-Ambrose et al., 2019 [45]	Strength + balance (home)	Usual care	Fall incidence (12 months)	↓ Falls	Reduction in recurrent falls
Li et al., 2018 [42]	Tai Ji Quan	Multimodal exercise	Falls (24 weeks)	↓ Falls	TJQ reduced falls by ~31%
Li et al., 2019 [46]	Tai Ji Quan	Stretching/MME	Injurious falls	↓ Injurious falls	Continued effect at follow-up
El-Khoury et al., 2015 [36]	Balance training	No exercise	Fall-induced injuries	↓ Moderate injuries	2-year benefit
Patil et al., 2015 [37]	Multimodal training	Usual activity	Falls (24 months)	↓ Falls	High adherence improved effect
Gawler et al., 2016 [39]	ProAct65+	Usual GP care	Falls	↓ Falls	Community-based fall reduction
Boongird et al., 2017 [41]	Home-based strength + balance	Usual care	Falls (12 months)	↓ Falls	Significant reduction
Waters et al., 2022 [58]	Otago	Education	Falls	↓ Falls	Effective in Native American elders
Allin et al., 2020 [51]	Slip-trip perturbation	Flexibility	Lab-induced falls	↓ Falls	Large reduction in slip/trip falls
Nørgaard et al., 2023 [59]	Treadmill perturbation	No training	Fall rates	↓ Falls	Strong improvement (lab)
Aviles et al., 2019 [44]	Trip-like treadmill	Tai Ji Quan	Reactive falls	↓ Falls	Superior reactive stability
Okubo et al., 2019 (RCT) [47]	Slip-trip RBT	Strengthening	Balance recovery/falls	↓ Falls	Task-specific improvements
LaStayo et al., 2017 [52]	Eccentric resistance	Traditional resistance	Falls	↔ No difference	Greater functional gains
Gianoudis et al., 2014 [54]	Multimodal power-based	Usual activity	Fall	↔ No reduction	Functional gains without fall reduction
Halvarsson et al., 2021 [55]	StayBalanced	Usual care	Falls	↓ Falls	Community rollout effective
Kulkarni et al., 2022 [57]	Group strength + balance	No exercise	Fall risk	↓ Fall risk	Reduced fear + risk
Costa et al., 2022 [56]	Balance circuit	Traditional exercise	Postural stability	↓ Instability	Indirect fall risk reduction
Mansfield et al., 2010 [34]	Compensatory stepping	Flexibility	Induced falls	↓ Falls	Better recovery responses
Lacroix et al., 2016 [38]	Supervised strength + balance	Unsupervised	Falls (indirect)	↓ Fall risk	Better muscle power/balance

↓ indicates a reduction in the reported outcome (e.g., falls, injurious falls, fall risk, or instability). ↔ indicates minimal, negligible, or no meaningful change compared with baseline or control conditions.

**Table 6:** Outcome Summary for Balance Performance.

Study	Intervention	Outcome Measure	Instrument Used	Effect Direction
Mansfield et al., 2010 [34]	Perturbation stepping & grasp training	Compensatory balance reactions	Force plates, motion analysis	↑ Compensatory balance
Stevens et al., 2013 [35]	ProAct65+ strength + balance	Functional balance	BBS	↑ Balance
El-Khoury et al., 2015 [36]	Long-term balance training	Balance stability	Clinical balance tests	↑ Balance
Patil et al., 2015 [37]	Multimodal exercise	Functional balance	SPPB balance	↑ Balance
Lacroix et al., 2016 [38]	Strength + balance	Static & dynamic balance	BBS, force plates	↑ Balance
Gawler et al., 2016 [39]	ProAct65+	Functional balance	BBS, TUG	↑ Balance
Trombetti et al., 2016 [40]	Music-based multitasking balance training	Gait & balance variability	GAITRite, BBS	↑ Balance stability
Boongird et al., 2017 [41]	Home-based strength + balance	Balance control	FES-I	↑ Balance confidence
Li et al., 2018 [42]	Therapeutic Tai Ji Quan	Functional balance	TUG, BBS	↑ Balance
Jansen et al., 2018 [43]	LiFE program (group vs. individual)	Functional balance	SPPB, TUG	↑ Balance
Aviles et al., 2019 [44]	Trip-like treadmill training	Reactive balance	Motion capture	↑ Reactive balance
Liu-Ambrose et al., 2019 [45]	Home-based strength + balance	Mobility & balance	SPPB, gait tests	↑ Balance & mobility
Li et al., 2019 [46]	Tai Ji Quan	Balance recovery & postural control	Clinical balance tests	↑ Reactive & functional balance
Okubo et al., 2019 (RCT) [47]	Slip-trip perturbation	Reactive balance	Slip-trip treadmill	↑ Reactive balance
Okubo et al., 2019 (Pilot RCT) [48]	Unpredictable slip-trip training	Biomechanical balance control	Vicon motion analysis	↑ Reactive balance
Arkkukangas et al., 2019 [49]	Otago + behavior support	Balance & adherence	BBS	↑ Balance
Cederbom & Arkkukangas, 2019 [50]	Long-term Otago	Balance & functional ability	Self-reported function tests	↑ Functional balance
Allin et al., 2020 [51]	Perturbation-based balance training	Reactive balance	Slip-trip treadmill	↑ Balance recovery
Bhasin et al., 2020 [53]	STRIDE multifactorial intervention	Functional mobility	Gait speed, mobility tests	↔ Minimal improvement in balance
Gianoudis et al., 2014 [54]	Multimodal power-based exercise (Osteo-cise)	Dynamic balance	Four Square Step Test, Sit-to-Stand	↑ Balance
LaStayo et al., 2017 [52]	Eccentric resistance training	Functional balance	Functional balance tests	↑ Balance
Halvarsson et al., 2021 [55]	StayBalanced	Functional balance	BBS	↑ Balance
Costa et al., 2022 [56]	Balance exercise circuit	Postural stability	Force platforms	↑ Balance
Kulkarni et al., 2022 [57]	Group exercise	Balance ability	BBS	↑ Balance
Waters et al., 2022 [58]	Otago	Functional balance	TUG	↑ Balance
Nørgaard et al., 2023 [59]	Treadmill perturbation	Reactive balance	Wearable sensors	↑ Balance recovery
Petrovic et al., 2024 [60]	Perturbation treadmill	Mobility & balance	SPPB, gait speed	↑ Balance & mobility

↑ indicates a significant or meaningful improvement in the reported outcome (e.g., balance, reactive balance, mobility, or confidence). whereas ↔ indicates no significant difference or no meaningful reduction compared with baseline or control conditions.

**Table 7:** Outcome Summary for Mobility/Functional Performance.

Study	Intervention	Outcome Measure	Instrument	Effect Direction
Liu-Ambrose et al., 2019 [45]	Strength + balance	Mobility	SPPB	↑ Mobility
Patil et al., 2015 [37]	Multimodal training	Mobility	SPPB	↑ Mobility
LaStayo et al., 2017 [52]	Eccentric resistance training	Functional mobility	Timed Up and Go, functional performance tests	↑ Mobility
Gianoudis et al., 2014 [54]	Multimodal power-based exercise (Osteo-cise)	Functional mobility	Timed Stair Climb, Sit-to-Stand	↑ Mobility
Petrovic et al., 2024 [60]	Perturbation treadmill	Gait speed, mobility	SPPB, gait tests	↑ Mobility
Costa et al., 2022 [56]	Balance circuit	Functional mobility	TUG	↑ Mobility
Kulkarni et al., 2022 [57]	Group exercise	Strength & mobility	TUG, Chair Stand	↑ Mobility
Waters et al., 2022 [58]	Otago	Mobility	TUG	↑ Mobility
Halvarsson et al., 2021 [55]	StayBalanced	Functional ability	Balance & mobility tests	↑ Mobility
Allin et al., 2020 [51]	Perturbation training	Functional stability	Slip/trip response	↑ Functional capacity

↑ indicates a significant or meaningful improvement in the reported outcome (e.g., balance, reactive balance, mobility, or confidence).

**Table 8:** Outcome Summary for Strength (Lower-Limb Strength).

Study	Intervention	Strength Measure	Instrument Used	Effect Direction
LaStayo et al., 2017 [52]	Eccentric resistance training	Lower-limb strength and power	Isokinetic dynamometry, functional strength tests	↑ Strength
Gianoudis et al., 2014 [54]	Multimodal high-velocity power training (Osteo-cise)	Lower-limb strength and power	Chair Stand Test, Timed Stair Climb	↑ Strength
Kulkarni et al., 2022 [57]	Group exercise	Lower-limb strength	Chair Stand Test	↑ Strength
Costa et al., 2022 [56]	Balance circuit	Muscle performance	Functional leg tests	↑ Strength
Patil et al., 2015 [37]	Multimodal training	Strength	Lower-limb dynamometry	↑ Strength
Lacroix et al., 2016 [38]	Strength + balance	Muscle power	Force platforms	↑ Power
Stevens et al., 2013 [35]	Strength + balance	Strength improvements	Functional tests	↑ Strength
Gawler et al., 2016 [39]	Strength + balance	Strength improvements	Functional tests	↑ Strength

↑ indicates a significant or meaningful improvement in the reported outcome (e.g., balance, reactive balance, mobility, or confidence).

## Data Availability

All data supporting the findings of this systematic review are derived from previously published studies. No new datasets were generated or analyzed during the current study. The full list of included articles, along with extraction sheets and methodological details, is available from the corresponding author upon reasonable request.

## Abbreviations

The following abbreviations are used in this manuscript:

BBS	Berg Balance Scale
OEP	Otago Exercise Program
PRISMA	Preferred Reporting Items for Systematic Reviews and Meta-Analyses
PROSPERO	International Prospective Register for Systematic Reviews
RCT	Randomized controlled trial
ROB-2	Cochrane Risk of Bias tool
SPPB	Short Physical Performance Battery
TUG	Timed Up and Go
LiFE	Lifestyle-Integrated Functional Exercise
STRIDE	Strategies to Reduce Injuries and Develop Confidence in Elders
IMU	Inertial Measurement Unit
